# Total elbow arthroplasty for distal humeral fractures in the elderly population: good functional outcomes and a high implant survival rate can be expected after surgery

**DOI:** 10.3389/fsurg.2026.1855944

**Published:** 2026-06-17

**Authors:** Giovanna Spina, Teresa Pugliese, Antonino Laganà, Lucrezia Moggio, Filippo Familiari, Giorgio Gasparini, Michele Mercurio

**Affiliations:** 1 Department of Orthopaedic and Trauma Surgery, “Magna Græcia” University, “Renato Dulbecco” University Hospital, Catanzaro, Italy; 2 Research Center on Musculoskeletal Health, MusculoSkeletalHealth@UMG, “Magna Græcia” University, Catanzaro, Italy; 3 Division of Orthopedics and Trauma Surgery, Polistena Hospital, Reggio Calabria, Italy; 4 Rehabilitation Unit, Ospedale degli Infermi, Biella, Italy

**Keywords:** arthroplasty, distal humeral fracture, distal humeral fracture treatment, elbow rehabilitation, elbow surgery, total elbow arthroplasty

## Abstract

**Introduction:**

Distal humeral fractures in elderly patients are difficult to treat due to poor bone quality and high complication rates. Total elbow arthroplasty (TEA) is an alternative for non-reconstructable fractures. This study aimed to evaluate long-term outcomes and implant survival of elderly subjects treated with linked TEA for acute distal humeral fractures.

**Methods:**

A retrospective study was conducted on data from patients who underwent TEA between January 2017 and December 2024 with fractures unsuitable for open reduction and internal fixation (ORIF). Clinical (range of motion, visual analogue scale, Mayo Elbow Performance Score, the Quick Disabilities of the Arm, Shoulder, and Hand score, and the American Shoulder and Elbow Surgeons score) and radiographic outcomes, complications, and implant survival were analysed.

**Results:**

Thirty-seven patients (mean age 80.4 ± 5.1 years) were followed for a mean of 107 ± 54 months. TEA permitted achievement of a good ROM (flexion 130 ± 8.16°; extension 19.8 ± 11.4°), and functional outcomes (VAS 3.3 ± 1.3, MEPS 83.5 ± 7.3, DASH 54 ± 4.4, ASES 78 ± 12.9) at the final follow-up. Revision rate was 11%, and 5-year implant survival was 94.2%. Comorbidities did not significantly influence clinical outcomes.

**Conclusion:**

TEA provides reliable long-term function and pain relief with acceptable complication rates in elderly patients with complex distal humeral fractures, supporting its use as a primary treatment in selected cases.

## 1 Introduction

Distal humeral fractures account for approximately 5%–7% of all humeral fractures and demonstrate a bimodal age distribution, with a second incidence peak in elderly individuals following low-energy trauma (1). In geriatric patients, osteoporosis, metaphyseal comminution, and articular fragmentation frequently compromise the possibility of achieving stable internal fixation (2). Open reduction and internal fixation (ORIF) has historically been considered the gold standard for displaced intra-articular distal humeral fractures (3). However, in elderly patients with poor bone stock, reported complication rates range between 20% and 40%, including non-union, fixation failure, implant-related irritation, and elbow stiffness (4). Even with modern locking plate technology, achieving durable fixation in severely osteoporotic bone remains technically demanding and often requires immobilization, which further increases stiffness risk (5). Total elbow arthroplasty (TEA) was initially developed for inflammatory arthritis but has progressively expanded to trauma indications, also considering possible preexisting elbow arthritis or associated ligamentous instability lesions (6). In complex AO Foundation and Orthopaedic Trauma Association (AO/OTA) partial or complete fractures not amenable to reconstruction, TEA offers several advantages: immediate stability, early mobilization, predictable pain relief, and independence from fracture healing biology (7, 8). However, TEA is a challenging decision-making process and procedure that requires experience in arthroplasty and managing related complications; stem loosening because of load transfer to the stem-cement-bone interface and polyethylene wear at the implant linkage should always be considered. A randomized controlled trial by McKee et al. (9) demonstrated superior short-term functional outcomes for TEA compared with ORIF in elderly patients with displaced intra-articular fractures. Subsequent series have confirmed satisfactory mid-term results (10–13). Despite increasing adoption, concerns remain regarding implant survivorship, periprosthetic radiolucency, polyethylene wear, infection, and mechanical failure (particularly in trauma-related indications, which historically show higher revision rates than inflammatory arthritis) (9, 14–16). Long-term data beyond five years in fracture-specific cohorts are still limited, and the influence of common geriatric comorbidities such as osteoporosis and rheumatoid arthritis (RA) on elbow functionality and implant stability remains incompletely understood.

Therefore, this study aimed to evaluate long-term clinical outcomes, radiographic findings, complication rates, and implant survival in elderly patients treated with linked TEA for acute distal humeral fractures. We hypothesized that TEA would provide durable functional outcomes with acceptable complication rates independent of major comorbidities.

## 2 Materials and methods

### 2.1 Study design and setting

This retrospective study followed the guidelines defined by the Strengthening the Reporting of Observational Studies in Epidemiology (STROBE) guidelines and checklist. The study protocol was approved by the Local Ethics Committee (Institutional Review Board approval was obtained from Mater Domini Ethics Committee. 14/2015), and the research was conducted in compliance with the Declaration of Helsinki. Written informed consent was obtained from all participants before their inclusion.

### 2.2 Participants

The study was conducted on data of patients who underwent TEA between January 2017 and December 2024. Inclusion criteria were: 1) age >65 years; 2) low-energy trauma; 3) 13 B or C AO-OTA fractures unsuitable for ORIF; 4) low-demand request; 5) acute fractures, osteoporotic fractures, fractures associated with elbow arthrosis or RA; 6) minimum 12-month follow-up. Exclusion criteria were: 1) elbow arthroplasty for other indications; 2) severe cognitive impairment; 3) previous elbow surgery (previous elbow arthroplasty or major reconstructive surgery, while minor or unrelated prior procedures were not considered exclusion criteria); 4) significant comorbidities precluding surgery; 5) substance abuse; 6) inability to complete the questionnaire. Demographic data collected included patient age at surgery, sex, body mass index (BMI), comorbidity (such as hypertension, cardiovascular diseases, diabetes mellitus, dyslipidemia, RA, osteoporosis under treatment), acetylsalicylic acid intake, upper limb dominance, and diagnosis of the affected elbow.

### 2.3 Preoperative assessment and fracture classification

Fracture patterns were classified according to the AO/OTA classification system based on standard anteroposterior and lateral elbow radiographs and CT obtained preoperatively, as described in previous radiographic studies of elbow fractures (17, 18).

### 2.4 Operative technique and postoperative protocol

All patients underwent surgery under general anesthesia combined with a brachial plexus block, positioned prone or lateral. Antibiotic prophylaxis was administered within 60 min before skin incision and continued for 24 h postoperatively, in accordance with International Guidelines for the prevention of surgical site infection (8, 19, 20). Linked prosthetic models fixed with antibiotic-loaded cement were always implanted [Coonrad- Morrey (Zimmer Inc, Warsaw, IN, USA) implant]. A Pooley approach (turn-down variant) was used in all cases, and the ulnar nerve was identified and protected (21). The humeral side is prepared first after carefully removing all fracture fragments, any hematoma or debris, and releasing the lateral and medial collateral ligaments. Then, the metaphyseal region was reshaped, either with a guide or freehand, according to the characteristics of the fracture. Using the most proximal portions of the coronoid and olecranon fossae as anatomical landmarks for determining implant length (22). The medullary canal was then identified, and the distal fractured segment was resected at the proximal margin of the lesion with saws and rongeurs to obtain a plane perpendicular to the humeral axis while maintaining cortical support on both columns (23). The canal was progressively prepared with increasing-sized rasps to ensure proper orientation of the humeral component relative to the supracondylar columns and the elbow's flexion-extension axis. The bony preparation is specific for each system used. Most components are stemmed and require preparation of the humeral and ulnar canals with rasps and broaches. Next, the canal is prepared to accept the stem, and the anterior cortex of the distal humerus is exposed for future contact with a bone graft placed behind the anterior flange of the humeral component. The ulnar canal is opened at the mid-portion of the trochlear notch, and the canal is prepared with right or left broaches. The components are then cemented in place with antibiotic-loaded polymethylmethacrylate, placing a bone graft between the anterior humeral cortex and the humeral flange. The components are then linked together. Linked semi-constrained implants were selected to provide stability in comminuted fractures.

Capitellar resection was not performed as a routine step of the procedure. Its indication was made intraoperatively on a case-by-case basis, primarily in the presence of severe capitellar comminution or when adequate implant positioning and joint stability could not be achieved due to local bone loss. Therefore, its use reflects fracture-specific surgical decision-making rather than a variation in the standardized surgical technique.

### 2.5 Postoperative protocol

Postoperative immobilization consisted of a brace for 15 days. The rehabilitation started on the first postoperative day with early passive and active-assisted range of motion exercises under the guidance of a physiotherapist, avoiding strengthening exercises. Moreover, the patient was advised not to lift more than one pound during the first three postoperative months, and not to lift more than five pounds thereafter as a lifelong restriction for the operated limb.

### 2.6 Clinical assessment

Clinical outcomes were assessed by measuring the intraoperative range of motion (ROM) and by evaluating pain using the Visual Analog Scale (VAS), which was recorded only at the last follow-up. Additionally, each patient completed a standardized set of validated elbow-specific outcome measures, including the Mayo Elbow Performance Score (MEPS) (24), the Quick Disabilities of the Arm, Shoulder, and Hand (quickDASH) score (25), and the American Shoulder and Elbow Surgeons (ASES) score (26). The MEPS is a 100-point scoring system that evaluates pain, motion, stability, and daily function, with higher scores indicating better performance. The quickDASH is a self-reported questionnaire also scored on a 100-point scale, where lower scores reflect less disability. Functional assessment further included evaluation of strength, range of movement, and performance in activities of daily living (27). Intraoperative ROM was measured using a goniometer. Muscle strength was assessed by comparing flexion and extension against counter-resistance with the elbow positioned at 90° of flexion on the operated limb relative to the contralateral side. Elbow stiffness was categorized as “severe” when the flexion–extension arc was ≤60° and “very severe” when ≤30° (28). Follow-up evaluations were scheduled at 1, 3, and 6 months, at 1 year, and at the most recent follow-up; however, elbow ROM was assessed intraoperatively and at the last follow-up, and VAS pain scores were collected exclusively at the last follow-up visit. Patient-reported satisfaction was recorded, and participants were asked whether they would undergo the same surgical procedure again.

### 2.7 Evaluation of complications and clinical outcome

Complications were categorized based on follow-up radiographs and medical chart reviews. Complications were categorized as follows: (1) Early complication; (2) Vascular (hematoma or need of blood transfusion); (3) Neurological (postoperative ulnar nerve and other nerve symptoms); (4) Mechanical (prosthetic loosening, metal breakage, and skin instrumentation irritation); (5) Infectious (superficial infection, deep infection, and wound complications); (6) Stiffness-related (heterotopic ossification or elbow stiffness) (29); (7) Death (even if unrelated with the procedure).

### 2.8 Radiological evaluation

All patients underwent radiographic evaluation at 1, 3, and 12 months postoperatively and at the time of the most recent follow-up. The radiographic evaluation was based on anteroposterior and lateral views of the elbow joint. Cement mantle quality for both the ulnar and humeral components was checked on immediate postoperative x-rays, and classified in 3 types, according to Morrey (PE-Morrey) criteria (30): grade 1 (optimal), defined as <1 mm radiolucency at the cement-bone interface with the cement mantle extending past the stem tip; grade 2 (borderline), characterized by 2 mm radiolucency with the cement reaching beyond the stem or <2 mm radiolucency without cement extension; and grade 3 (inadequate), indicated by >2 mm radiolucency without cement beyond the stem. Prosthetic stability was valued on x-ray, and scored 0 to 4, according to Morrey criteria (LE-Morrey) for evaluation at late follow-up (7): grade 0, <1 mm radiolucency affecting <50% of the interface; grade 1, 1 mm radiolucency affecting <50%; grade 2, >1 mm radiolucency affecting >50%; grade 3, >2 mm radiolucency across the full interface; grade 4, extensive osteolysis. Hinge polyethylene bushing wear was evaluated on final follow-up x-rays using the Lee and Morrey system (31), and categorized as: type 1 (normal), with ulnohumeral coronal angle below 3.5°; type 2 (partial wear), with bushing angulation from 3.5° to 5°; type 3 (full wear), with angulation greater than 5°. Anterior-posterior and lateral x-rays were examined for periarticular heterotopic ossification according to the Hastings and Graham classification (32). Two Authors, unaware of the patients' clinical features, reviewed the radiographic studies.

### 2.9 Statistical analysis

Continuous variables were described as mean ± standard deviation (SD) and median with interquartile range (IQR), whereas categorical variables were reported as absolute frequencies and percentages. The distribution of baseline data was assessed using the Shapiro–Wilk test. Given the non-normality observed in some variables, non-parametric statistical tests were used for all inferential analyses. Longitudinal differences between repeated measurements of the same parameter (for example, joint ROM at different follow-up intervals) were analyzed using the Friedman test, with subsequent pairwise *post-hoc* analyses performed using the Wilcoxon test with Bonferroni correction. Comparisons between two independent groups were carried out with the Mann–Whitney test for continuous variables, while Fisher's exact test was used for categorical variables with low frequencies. Correlations between continuous variables were assessed using Spearman's correlation coefficient (*ρ*). To evaluate the influence of multiple predictors on functional outcomes or final ROM, multivariable linear regression models were constructed. The survival of the prosthetic implant was analyzed with Kaplan–Meier curves. For all statistical tests, the level of significance was set at *p* < 0.05. Statistical analysis was performed using PAleontological STatistics (PAST) version 4.03, developed at the Natural History Museum, University of Oslo (Norway).

## 3 Results

**Figure 1:**
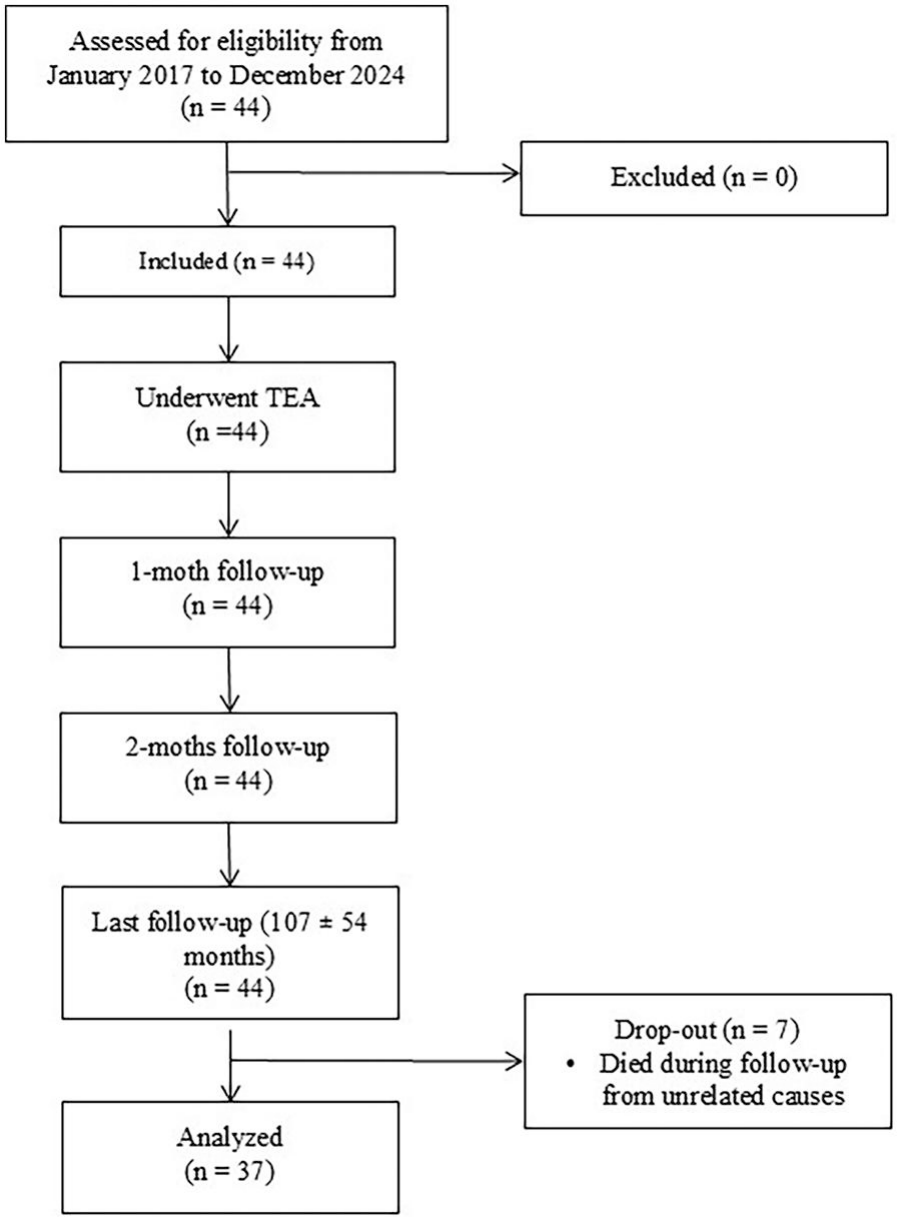
STROBE flow chart.

This study included data from 44 consecutive patients. Seven patients died during follow-up from unrelated causes, leaving 37 available for final analysis. Thus, a total of 37 patients who underwent TEA were included and evaluated at the last time after a mean follow-up of 107 ± 54 months (Figure 1). The mean age was 80.4 ± 5.1 years, with a median of 80 years (IQR 78–85), while the mean BMI was 29.3 ± 4.9 kg/m² (median 29; IQR 26–32). The sample consisted predominantly of women (*n* = 35, 94.5%). The operated limb was the right arm in 21 patients (56.7%), which corresponded to the dominant limb in 20 cases (95.2%). The main comorbidities were arterial hypertension in 23 patients (62.1%), cardiovascular disease in 2 (5.4%), diabetes mellitus in 6 (16.2%), dyslipidemia in 6 (16.2%), RA in 6 (16.2%), and treated osteoporosis in 14 patients (37.8%). ASA classification was I in 5 patients (13.5%), II in 24 (64.9%), and III in 8 (21.6%). The mean postoperative length of stay was 3.9 ± 1.1 days (median 3; IQR 3–5). The demographic characteristics are depicted in Table 1.

**Table 1:** Demographic characteristics of included subjects.

Patient ID	Sex	Age	BMI	Dominant limb	HTN	CVD	DM	DLP	ASA Classification	RA	OP	Previous elbow surgery	LOS (days)
1	F	82	22	R	NO	NO	NO	NO	2	NO	NO	NO	4
2	F	74	28	R	YES	NO	NO	NO	3	NO	NO	NO	3
3	F	64	28	R	YES	YES	YES	YES	3	YES	NO	YES	3
4	F	87	30	R	YES	NO	0	NO	3	NO	NO	NO	3
5	F	73	26	R	YES	NO	0	NO	2	NO	NO	NO	3
6	F	79	33	R	YES	NO	0	YES	2	NO	YES	NO	3
7	F	73	34	R	NO	NO	0	NO	1	NO	YES	NO	3
8	F	85	29	R	NO	NO	0	YES	3	YES	NO	NO	4
9	F	77	31	R	NO	NO	0	NO	2	NO	YES	NO	3
10	F	77	31	R	YES	NO	0	NO	2	NO	NO	NO	3
11	F	88	33	R	YES	NO	0	NO	2	NO	NO	NO	5
12	M	86	22	L	YES	NO	0	NO	2	YES	NO	NO	5
13	F	88	23	R	NO	NO	0	NO	2	NO	YES	NO	5
14	F	79	41	R	YES	NO	0	NO	2	YES	YES	NO	3
15	F	75	28	R	NO	NO	0	NO	2	NO	NO	NO	3
16	F	86	31	R	YES	NO	0	NO	2	NO	YES	NO	7
17	F	85	30	R	YES	NO	0	NO	1	NO	YES	NO	5
18	F	85	29	R	YES	NO	0	NO	2	NO	NO	NO	4
19	F	79	35	R	YES	NO	YES	YES	2	NO	NO	NO	3
20	F	75	29	R	YES	NO	YES	YES	3	NO	NO	NO	3
21	F	78	25	L	YES	NO	NO	NO	2	NO	NO	NO	3
22	F	79	24	R	NO	NO	NO	NO	1	NO	NO	NO	5
23	F	88	39	R	NO	NO	NO	NO	1	NO	NO	NO	3
24	F	78	25	R	NO	NO	NO	NO	1	YES	YES	NO	3
25	F	79	26	R	YES	NO	NO	NO	2	NO	NO	NO	3
26	F	88	31	R	YES	NO	NO	NO	2	NO	YES	NO	3
27	F	85	28	R	NO	NO	NO	YES	2	NO	NO	NO	5
28	F	82	29	L	YES	NO	NO	NO	2	NO	NO	NO	5
29	F	79	36	R	NO	YES	NO	NO	3	NO	NO	NO	5
30	F	80	37	R	NO	NO	NO	NO	3	YES	YES	NO	4
31	F	82	26	R	YES	NO	NO	NO	2	NO	YES	NO	3
32	F	83	26	R	YES	NO	NO	NO	2	NO	YES	NO	3
33	F	79	30	R	YES	NO	YES	NO	2	NO	NO	NO	7
34	F	81	28	R	YES	NO	YES	NO	3	NO	NO	NO	5
35	F	78	27	R	YES	NO	NO	NO	2	NO	NO	NO	4
36	M	82	29	R	NO	NO	YES	NO	2	NO	YES	NO	5
37	F	80	23	R	NO	NO	NO	NO	2	NO	YES	NO	5

Data are presented as medians. M, male; F, female; BMI, body mass index; L, left; R, right; HTN, hypertension; CVD, cardiovascular disease; DM, diabetes mellitus; DLP, dyslipidaemia; ASA, American Society of Anaesthesiologists; RA, rheumatoid arthritis; OP, osteoporosis; LOS, length of stay.

Early complications occurred in 2 patients (5.4%), postoperative hematomas were observed in 5 patients (13.5%), and neurological complications occurred in 3 (8.1%). Blood transfusion was required in 10 patients (27%). Intraoperative range of motion showed a mean flexion of 136.2 ± 8° and an extension of 9 ± 5.5°. A summary of postoperative complications is presented in Table 2.

**Table 2:** Early and late postoperative complications.

Patient ID	**Early complication**	**BT**	**Hematoma**	**Neurological complication**	**Implant loosening**	**Polyethylene wear**	**Heterotopic ossification**	**Radiolucent lines**
1	NO	NO	NO	NO	NO	NO	YES	NO
2	NO	YES	NO	NO	NO	NO	YES	NO
3	NO	NO	NO	NO	NO	NO	YES	< 1mm
4	NO	YES	YES	NO	NO	NO	NO	NO
5	NO	NO	NO	NO	NO	NO	YES	<1 mm
6	NO	YES	YES	NO	NO	NO	YES	>2 mm
7	NO	NO	NO	NO	NO	NO	NO	NO
8	NO	NO	NO	NO	NO	NO	NO	NO
9	NO	NO	NO	NO	NO	NO	NO	NO
10	NO	NO	NO	NO	NO	NO	YES	NO
11	NO	NO	NO	NO	NO	NO	YES	<1 mm
12	YES	YES	NO	NO	YES	NO	YES	>2 mm
13	NO	NO	NO	YES	NO	NO	NO	NO
14	NO	NO	NO	NO	NO	NO	YES	NO
15	NO	NO	NO	NO	NO	NO	NO	NO
16	NO	NO	NO	YES	NO	NO	YES	NO
17	NO	NO	NO	NO	NO	NO	YES	NO
18	NO	NO	NO	NO	NO	NO	YES	NO
19	NO	NO	YES	NO	NO	NO	YES	<1 mm
20	NO	NO	NO	NO	NO	NO	NO	<1 mm
21	NO	NO	NO	YES	NO	NO	YES	NO
22	NO	YES	NO	NO	NO	NO	YES	NO
23	NO	YES	NO	NO	NO	NO	NO	NO
24	NO	NO	YES	NO	NO	NO	NO	<1 mm
25	NO	NO	NO	NO	NO	NO	NO	NO
26	NO	YES	NO	NO	YES	NO	YES	NO
27	NO	NO	NO	NO	NO	NO	YES	NO
28	NO	YES	NO	NO	NO	YES	YES	NO
29	NO	NO	NO	NO	NO	NO	NO	NO
30	NO	YES	YES	NO	NO	NO	YES	NO
31	NO	NO	NO	NO	YES	NO	NO	>2 mm
32	NO	NO	NO	NO	NO	NO	YES	<1 mm
33	NO	NO	NO	NO	NO	NO	YES	NO
34	NO	NO	NO	NO	NO	YES	YES	NO
35	YES	NO	NO	NO	NO	NO	YES	NO
36	NO	NO	NO	NO	NO	YES	NO	NO
37	NO	YES	NO	NO	YES	NO	NO	NO

Data are presented as medians. BT, blood transfusion.

**Figure 2:**
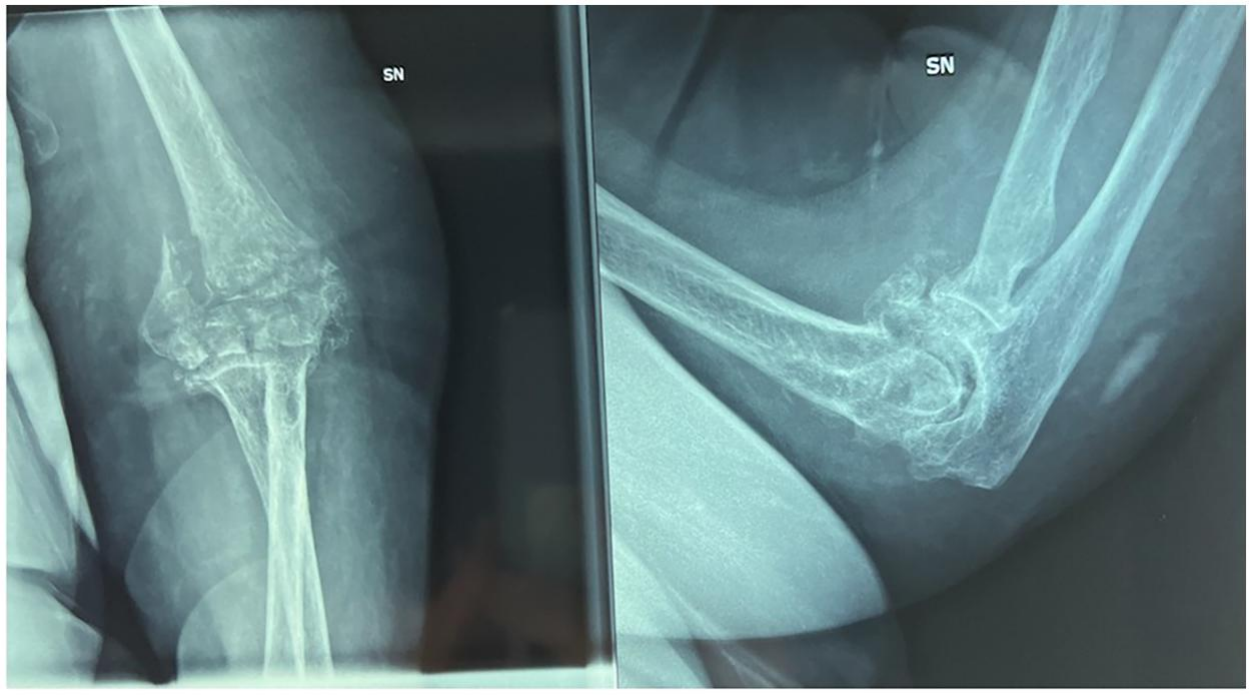
Preoperative anteroposterior and lateral radiographs of the elbow.

**Figure 3:**
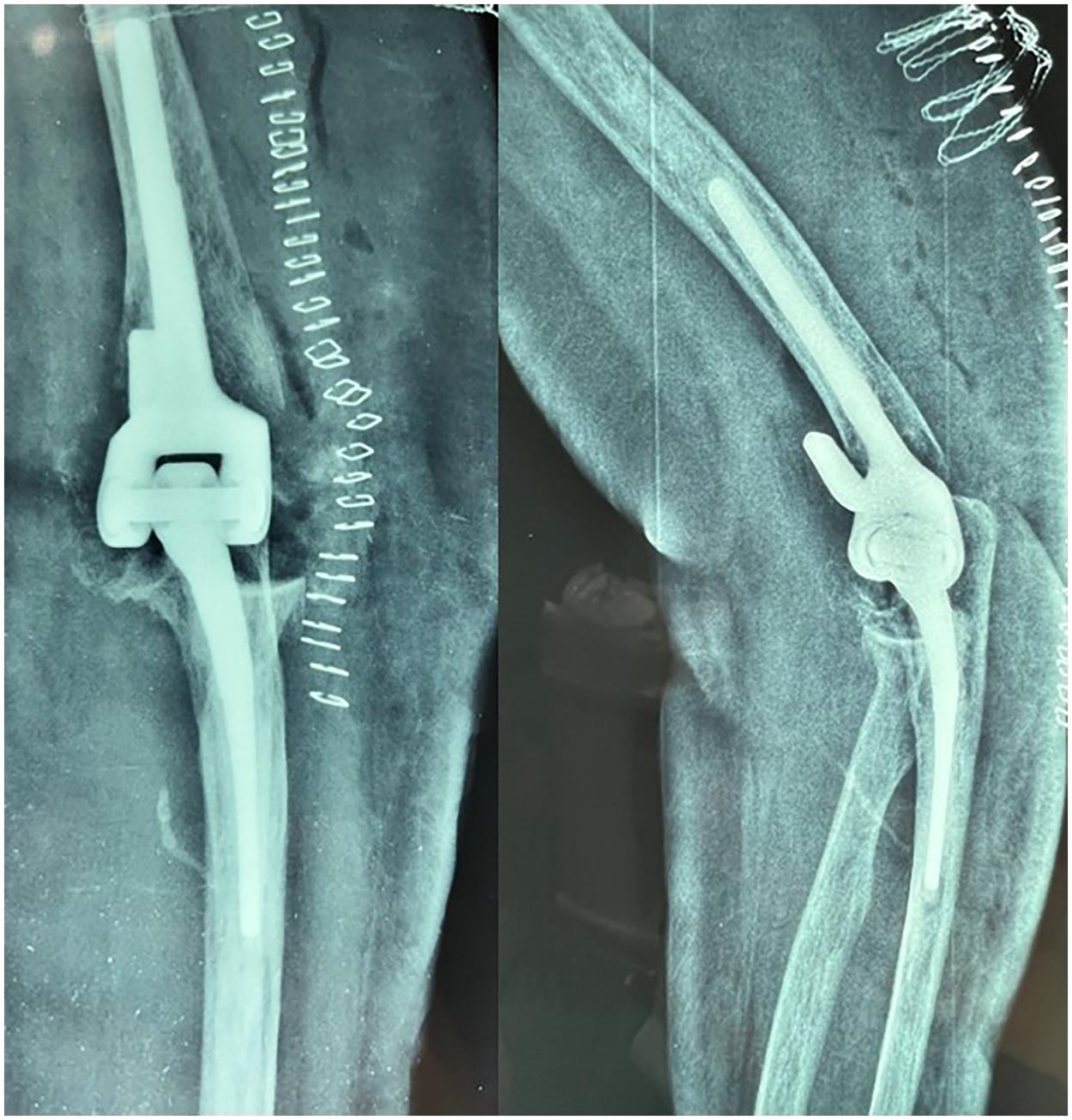
Postoperative anteroposterior and lateral radiographs of the elbow showing a total elbow replacement with well-aligned components.

All 37 patients underwent cemented TEA. Stem sizes ranged from XS to M, with S being the most frequent (18/37, 48.6%), and ulnar component sizes ranged from XS to S, with XS being most common (16/37, 43.2%). Capitellum resection was performed in 9 patients (24.3%), and bone grafting was used in all cases (100%). The surgical details are summarized in Table 3, and the radiological images are depicted in Figures 2, 3.

**Table 3:** Surgical procedures and prosthesis details.

Patient ID	Type of intervention	Cemented prosthesis	Stem size (1–4 inch)	Ulnar component size (1–3 inch)	Capitellum Resection	Bone graft
1	TEA	YES	S	XS	NO	YES
2	TEA	YES	XS	XS	NO	YES
3	TEA	YES	M	M	NO	YES
4	TEA	YES	S	XS	NO	YES
5	TEA	YES	S	S	YES	YES
6	TEA	YES	XS	XS	NO	YES
7	TEA	YES	S	XS	NO	YES
8	TEA	YES	S	S	NO	YES
9	TEA	YES	S	XS	NO	YES
10	TEA	YES	XS	XS	NO	YES
11	TEA	YES	XS	XS	NO	YES
12	TEA	YES	XS	XS	NO	YES
13	TEA	YES	XS	XS	NO	YES
14	TEA	YES	XS	XS	YES	YES
15	TEA	YES	S	S	NO	YES
16	TEA	YES	S	S	NO	YES
17	TEA	YES	S	S	NO	YES
18	TEA	YES	S	S	NO	YES
19	TEA	YES	S	XS	NO	YES
20	TEA	YES	M	M	YES	YES
21	TEA	YES	S	XS	YES	YES
22	TEA	YES	S	2	YES	YES
23	TEA	YES	XS	XS	NO	YES
24	TEA	YES	XS	XS	NO	YES
25	TEA	YES	XS	XS	YES	YES
26	TEA	YES	XS	XS	NO	YES
27	TEA	YES	S	S	NO	YES
28	TEA	YES	S	S	NO	YES
29	TEA	YES	S	S	NO	YES
30	TEA	YES	S	XS	NO	YES
31	TEA	YES	M	M	NO	YES
32	TEA	YES	M	M	YES	YES
33	TEA	YES	S	XS	YES	YES
34	TEA	YES	XS	XS	NO	YES
35	TEA	YES	XS	XS	NO	YES
36	TEA	YES	S	XS	NO	YES
37	TEA	YES	S	S	YES	YES

TEA, total elbow arthroplasty.

Concerning the radiological characteristics, cement mantle quality was grade 0 in 62.2%, grade 1 in 24.3%, and grade 3 in 13.5%. Radiographic loosening was absent in 67.6% of cases, while grade 1 loosening was observed in 24.3%, grade 2 in 5.4%, and grade 3 in 2.7%. Polyethylene bushing wear was predominantly mild, with grade 1 in 91.9% of patients; higher-grade wear was uncommon (grade 2: 2.7%; grade 3: 5.4%). Overall, most patients showed adequate cement mantle quality, low rates of radiographic loosening, and minimal polyethylene wear (see Table 4 for further details).

**Table 4:** Radiographic characteristics of included patients.

Patient ID	Cement mantle quality	Radiographic loosening	Polyethylene bushing wear	Heterotopic ossification
1	3	0	1	1
2	1	0	1	1
3	1	1	1	1
4	1	0	1	-
5	1	1	1	1
6	1	1	1	2
7	1	0	1	-
8	1	0	1	-
9	2	0	1	-
10	1	0	1	1
11	2	2	1	1
12	1	1	1	1
13	2	0	1	-
14	1	0	1	1
15	2	0	1	-
16	2	0	1	1
17	2	0	1	1
18	2	0	1	1
19	1	3	1	2
20	1	1	1	-
21	2	0	1	1
22	1	0	1	1
23	1	0	1	–
24	1	1	1	–
25	1	0	1	–
26	3	0	1	1
27	2	0	1	2
28	1	0	3	1
29	2	0	1	-
30	1	0	1	1
31	3	1	1	-
32	2	2	1	1
33	1	0	1	1
34	1	0	3	2
35	1	0	1	1
36	1	0	2	–
37	3	0	1	–

Regarding the postoperative satisfaction, only 4 patients out of 37 (10.8%) stated that they would not undergo the same surgical procedure. These cases were primarily associated with lower functional scores and/or postoperative complications.

### 3.1 Clinical results

At 30 days, median ROM was 126.2° in flexion (IQR 120–130) and 20.2° in extension (IQR 10–20), while pronation and supination were 73.2° and 71.7°, respectively. At 60 days, a further improvement was observed (flexion 132.9°, extension 13.7°, pronation 77°, supination 70.9°). After a mean of 6.7 ± 3.2 months of rehabilitation (median 6; IQR 4–9), patients showed a mean flexion of 130 ± 8.16°, extension 19.8 ± 11.4°, pronation 78.6°, and supination 78.9°. Extension ROM improved significantly over time [Friedman *χ*^2^(3) = 17.33, *p* < 0.001]. *post-hoc* analyses showed significant differences between intraoperative and 30 days (*p* < 0.001), 60 days (*p* = 0.043), and final follow-up (*p* < 0.001), as well as between 30 and 60 days (*p* = 0.001) and between 60 days and final follow-up (*p* < 0.001). Flexion also showed a significant improvement [Friedman *χ*^2^(3) = 23.53, *p* < 0.01], with significant differences between intraoperative and 30 days (*p* < 0.001), intraoperative and final follow-up (*p* = 0.003), and between 30 and 60 days (*p* < 0.001). Pronation and supination also improved significantly over time, although *post-hoc* analysis identified significant differences only between some intervals (Table 1).

Functional scores at last follow-up were: VAS 3.3 ± 1.3, MEPS 83.5 ± 7.3, DASH 54 ± 4.4, ASES 78 ± 12.9. Overall, 34 patients (91.8%) achieved good or excellent MEPS scores (>75).

Among the 37 patients, 4 (10.8%) underwent reintervention: 1 (2.7%) for a periprosthetic fracture and 3 (8.1%) for aseptic loosening requiring revision of the ulnar component. Heterotopic ossification was observed in 23 patients (62.2%), and polyethylene wear in 3 patients (8.1%); however, none of these cases required reoperation.

Functional outcomes are summarized in Table 5.

**Table 5:** Functional outcomes of the included subjects.

Patient ID	Intra operative extension (degrees)	Intra operative flexion (degrees)	Extension at 30 days (degrees)	Flexion at 30 days (degrees)	Pronation at 30 days (degrees)	Supination at 30 days (degrees)	Extension at 60 days (degrees)	Flexion at 60 days (degrees)	Pronation at 60 days (degrees)	Supination at 60 days (degrees)	Extension at follow-up (degrees)	Flexion at follow-up (degrees)	Pronation at follow-up (degrees)	Supination at follow-up (degrees)	Post operative rehabilitation duration (months)	MEPS	VAS	DASH (%)	ASES
1	10	140	25	120	80	50	20	140	80	80	30	130	80	80	5	88	4	55	75
2	20	140	10	120	80	80	10	140	80	80	10	140	80	80	3	85	4	52	78
3	10	140	30	120	0	0	25	120	50	20	20	130	30	40	12	62	5	60	65
4	0	140	10	120	80	80	10	130	80	80	20	130	80	80	5	89	2	48	92
5	20	120	10	130	80	80	0	140	80	80	20	130	80	80	3	62	5	55	70
6	10	145	20	130	80	80	20	130	80	80	20	130	80	80	9	85	3	57	78
7	10	130	10	140	80	80	0	140	80	80	10	130	80	80	9	83	3	55	80
8	10	130	20	110	80	80	20	120	80	80	20	120	80	80	12	85	4	55	75
9	10	140	20	130	80	80	10	140	80	80	10	140	80	80	9	85	2	44	96
10	10	140	40	130	80	80	30	130	80	80	30	130	80	80	4	85	2	53	87
11	20	110	10	140	80	80	10	140	80	80	20	140	80	80	6	85	4	55	75
12	10	130	10	140	80	80	20	140	80	80	60	110	80	80	6	76	6	65	55
13	10	140	0	140	80	80	5	130	80	80	10	120	80	80	9	91	2	52	88
14	0	140	20	140	80	80	20	140	80	80	30	130	80	80	3	80	4	54	76
15	10	140	10	110	80	80	10	110	80	80	30	110	80	80	9	92	2	48	92
16	10	130	60	130	80	45	60	130	80	45	50	130	80	80	12	92	3	55	80
17	0	140	0	140	80	80	5	140	80	80	30	110	80	80	4	86	4	55	75
18	10	140	10	120	80	80	5	140	80	80	20	130	80	80	12	80	4	56	74
19	10	115	0	140	80	80	0	140	80	80	20	130	80	80	6	85	4	56	74
20	5	140	0	130	80	80	0	130	80	80	0	140	80	80	3	82	3	50	85
21	10	130	20	120	50	0	20	130	60	0	20	130	80	80	3	94	2	44	96
22	10	140	20	130	80	80	10	140	80	80	10	140	80	80	5	77	7	55	60
23	10	140	10	130	80	80	10	140	80	80	10	140	80	80	12	84	3	55	80
24	10	140	10	130	80	80	0	140	80	80	5	140	80	80	9	89	3	53	82
25	20	140	10	130	80	80	0	140	80	80	10	140	80	80	9	77	6	60	60
26	0	140	20	120	60	80	10	135	70	80	10	130	80	80	9	82	4	58	72
27	0	150	20	120	60	80	20	120	80	80	20	130	80	80	6	85	4	53	30
28	0	140	20	130	80	80	30	130	80	80	30	130	80	80	3	76	5	60	65
29	10	130	20	130	80	80	15	130	80	80	20	130	80	80	3	91	1	48	97
30	10	140	20	120	50	0	20	130	60	0	20	130	80	80	3	88	3	48	87
31	10	140	10	130	80	80	0	140	80	80	20	130	80	80	6	80	2	50	90
32	10	140	10	130	80	80	10	140	80	80	10	140	80	80	6	92	2	55	85
33	10	140	40	130	80	80	10	135	70	80	10	130	80	80	5	72	3	55	80
34	10	130	60	115	80	80	20	120	80	80	20	120	80	80	9	86	2	55	85
35	10	140	35	135	30	80	20	120	80	80	20	130	80	80	3	80	2	57	83
36	0	130	40	80	60	80	15	130	80	80	20	130	80	80	6	90	3	55	80
37	10	140	70	110	80	80	20	130	60	0	20	130	80	80	12	89	1	60	85
	9.05 (5.5)	136.2 (8)	20,2 (16.7)	126.2 (11.8)	73.2 (16.8)	71.7 (22.8)	13.7 (11.6)	132.9 (8)	77 (7.4)	70.9 (24.1)	19.8 (11.4)	130 (8.1)	78.6 (8.2)	78.9 (6.5)	6.7 (3.2)				

Data are reported ad mean (SD). MEPS, Mayo Elbow Performance Score; VAS, visual analog scale; DASH, Disability of the Arm, Shoulder and Hand; ASES, American Shoulder and Elbow Surgeons Shoulder Score.

### 3.2 Inferential analysis—group comparisons

No significant differences were found in extension ROM (*U* = 72.5; *p* = 0.37) or flexion ROM (*U* = 71.5; *p* = 0.32), MEPS (*U* = 69.5; *p* = 0.34), or VAS (*U* = 51.5; *p* = 0.08) between patients with or without RA. Similar results were observed in patients with or without osteoporosis (extension ROM: *U* = 140.5; *p* = 0.5; flexion ROM: *U* = 158.5; *p* = 0.9; MEPS: *U* = 100; *p* = 0.05; VAS: *U* = 108; *p* = 0.09). Likewise, the presence of early complications did not significantly affect postoperative functional parameters. Significant differences were observed in radiolucency scores between patients with and without osteoporosis (*p* = 4.75 × 10⁻⁶) and between patients with and without RA (*p* = 0.024), suggesting that these comorbidities are associated with increased periprosthetic radiographic changes. In contrast, no significant associations were found between osteoporosis or RA and the occurrence of heterotopic ossification (*p* = 0.70 and *p* = 1.0, respectively). Similarly, implant loosening, which occurred in three cases and was aseptic, showed no significant correlation with either osteoporosis (*p* = 0.14) or RA (*p* = 0.42).

### 3.3 Inferential analysis—correlations

Spearman correlations did not show significant associations between age and postoperative ROM, nor with MEPS, DASH, or VAS, which was confirmed also after stratification by age groups (<75, 76–79, 80–84, >85 years). Similarly, no significant correlations were found between length of hospital stay and postoperative parameters, either in the overall sample or in stratified subgroups (3, 4, 5, and 7 days).

### 3.4 Associations with complications

No significant associations were observed between complications and the presence of RA (*p* = 0.25), osteoporosis (*p* = 1.00), or age groups (*p* = 0.13), using Fisher's exact test.

### 3.5 Predictive models

The multivariable linear regression model for final MEPS, with age, total intraoperative ROM, and months of rehabilitation as predictors, explained approximately 18.5% of the variability in scores [R^2^ = 0.185; F(3,33) = 2.50; *p* = 0.077], showing a significant association only with age (*β* = 0.593; *p* = 0.013). The model for final DASH explained a limited proportion of variance (R^2^ = 0.087; *p* = 0.386), with no significant predictors. Finally, the linear regression model for final extension ROM, with intraoperative extension, age, complications, and RA as predictors, did not yield significant results (R^2^ = 0.127; *p* = 0.346).

In the multivariable linear regression analysis, including rehabilitation duration, and RA, only age showed a statistically significant association with final MEPS (*p* = 0.013).

**Figure 4:**
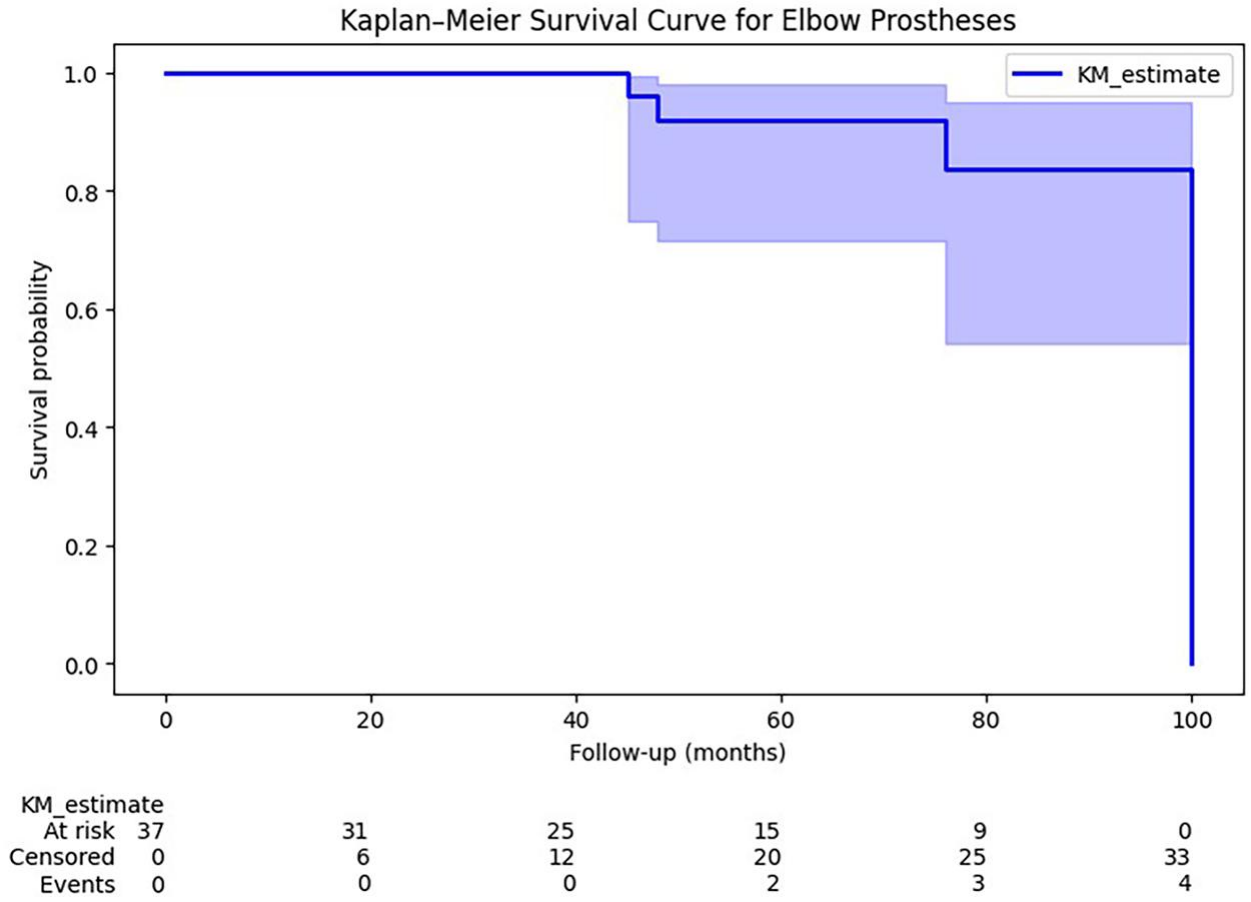
Kaplan–meier survival curve for elbow protheses.

Implant survival was evaluated using the Kaplan–Meier method. During the follow-up period, 5 patients required revision surgery. At 5 years (60 months), the estimated implant survival was 94.2% (95% CI: calculated from KM analysis) (Figure 4). Patients who did not experience implant failure were censored at their last follow-up.

## 4 Discussion

This retrospective study aimed to evaluate the long-term clinical and radiographic outcomes, complications, and implant survivorship of TEA performed for acute distal humeral fractures in elderly patients. At a mean follow-up of 107 months, TEA provided durable functional recovery, satisfactory pain control, and 94% implant survival rate, with good-to-excellent MEPS results in the majority of patients and a low revision surgery rate (11%). These findings support the role of TEA as a reliable primary treatment option in carefully selected low-demand geriatric patients with complex articular fractures not amenable to ORIF.

Management of distal humeral fractures in osteoporotic elderly patients remains controversial. While ORIF preserves native anatomy, multiple studies have reported high mechanical failure rates in elderly individuals with poor bone stock, often exceeding 20%–40%, even with modern locking constructs (33, 34). In contrast, TEA bypasses the biological dependence on fracture healing and allows early mobilization. The randomized controlled trial by McKee et al. (9) demonstrated superior short-term functional outcomes of TEA compared with ORIF in elderly patients with displaced articular fractures, with more predictable pain relief and earlier return to function. Subsequent long-term series, including those by Barco and colleagues (11), reported mean MEPS values above 85 at extended follow-up, with revision rates ranging between 10% and 18% in fracture-related TEA. In our cohort, the mean MEPS of 83.5 and revision rate of 11% compare favorably with previously published fracture-specific series. The approximately 95% implant survival observed at nine years suggests that contemporary cementing techniques and semi-constrained linked designs may contribute to improved durability compared with earlier reports. This improvement may reflect advances in implant design, particularly the use of semi-constrained linked systems, as well as refinements in cementation techniques and perioperative management (35). Additionally, the relatively low functional demands of this population may contribute to reduced mechanical stress at the bone–cement–implant interface, potentially enhancing implant longevity. In support of this claim, age was the only variable significantly associated with final MEPS in multivariable analysis (*β* = 0.593; *p* = 0.013), although the overall predictive capacity of the model was limited (*R*^2^ = 0.185). Several recent studies have reported favorable short- and mid-term outcomes with TEA for acute distal humeral fractures in elderly patients. Jost et al. showed comparable medium-term to long-term results in patients with RA who had suffered displaced distal humeral fractures and had been treated by ORIF or TEA (MEPS, 93 vs. 96 points; flexion, 130° vs. 133°, respectively) (36). The authors concluded that ORIF was successful when there was mild arthritis; however, for severe arthritis, TEA was preferable (37). In our study, satisfactory functional outcomes were observed at a mean follow-up of 107 months. Excellent/good MEPS scores (>75) in over 80% of cases confirm TEA's predictability for pain relief and daily function, aligning with series such as Barco et al. (MEPS 90.5 at 10 years) (11) and Dumoulin et al. (MEPS 85.3; quickDASH 28.1) (28). Although formal MCID thresholds were not assessed in this paper, reported improvements in MEPS, quickDASH, and range of motion exceed commonly reported minimal clinically important differences in elbow outcome literature (approximately 10–15 points for MEPS and quick-DASH, and 30° for flexion–extension arc), suggesting clinically meaningful functional recovery (38, 39).

Seven patients in our series died within 5 years after surgery from unrelated causes, consistent with the advanced age of candidates selected for TEA; therefore, fewer patients reached the timeframe where mechanical complications typically emerge. The progressive and sustained improvement in range of motion observed in our cohort highlights one of the key advantages of TEA in this setting: immediate mechanical stability combined with early mobilization. In elderly patients, prolonged immobilization following ORIF may result in stiffness and functional decline, particularly in the presence of comorbidities. From a practical standpoint, comorbidities had little impact, underscoring the importance of careful patient selection, counseling regarding lifelong lifting restrictions, and adherence to standardized postoperative rehabilitation.

Regarding the radiological aspects, our findings indicate that osteoporosis and RA may contribute to increased periprosthetic radiolucencies, potentially reflecting adaptive bone remodeling rather than true mechanical failure, and did not translate into clinically relevant loosening or heterotopic ossification. Thus, the absence of correlation between these radiographic findings and clinical outcomes in our cohort suggests that caution is warranted when interpreting radiolucent lines in elderly patients, as they may not necessarily indicate impending implant failure. Prospective studies with larger cohorts and longer follow-up are warranted to clarify these relationships and optimize management strategies for high-risk patients. Minimal radiographic loosening (Morrey PE/LE mostly grades 0–1) and low polyethylene wear (around 5% type 3) likely resulted from rigorous cementation with antibiotic-loaded cement and systematic bone grafting. No complications correlated with comorbidities (*p* > 0.05, Fisher's exact test), though patient counseling on permanent restrictions (no lifting >5 kg) remains essential. This aligns with previous observations that mild radiographic changes in elderly bone may not compromise function (9). However, continued surveillance remains warranted, particularly beyond the 10-year threshold when polyethylene wear and progressive osteolysis may become clinically relevant. Future survival analyses incorporating time-to-event modelling will help clarify the long-term behaviour of these implants in fracture-related indications.

This study has several strengths. First, it represents a consecutive single-centre cohort with standardized surgical technique and postoperative management. Second, the long-term follow-up, approaching nine years, provides meaningful insight into implant durability in a fracture-specific elderly population. Third, a comprehensive clinical and radiographic evaluation allowed correlation between functional outcomes and imaging findings. Finally, multivariable regression analysis was performed to explore potential predictors of outcome, increasing the robustness of the findings.

However, several limitations must be acknowledged. The retrospective design inherently introduces potential selection bias and limits causal inference, and the relatively small sample size reduces statistical power and may explain the limited significance of multivariable models; the regression model should be interpreted cautiously given the low R^2^ value (0.185) and the risk of overfitting. Second, follow-up duration was variable, with a minimum of 12 months, which may have influenced the assessment of long-term functional outcomes and complication rates, despite the availability of extended follow-up data in a subset of patients. Third, the absence of a control group treated with open reduction and internal fixation management precludes direct comparative conclusions regarding the relative effectiveness of different treatment strategies. As a result, the observed outcomes should be interpreted as procedure-specific rather than comparative. Additionally, potential selection bias cannot be excluded, as TEA was performed in patients deemed unsuitable for reconstruction, which may have influenced both baseline characteristics and postoperative expectations. Moreover, institutional data on alternative treatments were not available, preventing precise quantification of case selection. From a technical perspective, outcomes may also be influenced by surgical expertise and standardized protocols, including careful identification and protection of the ulnar nerve, meticulous cementing technique, and structured postoperative rehabilitation combined with patient education. These factors may limit the reproducibility of the results in lower-volume centers. Finally, although mid- to long-term implant survivorship was favorable, the study was not specifically designed to evaluate late mechanical failure or polyethylene bushing wear in linked semiconstrained implants beyond 10 years. Given the limited sample size relative to the number of predictors, all multivariable analyses should be considered exploratory and hypothesis-generating. Prospective, multicenter randomized studies with standardized follow-up and contemporary outcome measures are therefore needed to confirm these findings, better define indications, and assess long-term implant performance.

In the context of an aging population with increasing fragility fractures, the choice of definitive and reliable treatment becomes particularly important. Notably, seven patients in our cohort died during follow-up from unrelated causes, underscoring the frailty of this population. In such patients, a procedure that minimizes the likelihood of secondary surgery and provides predictable pain relief may offer significant clinical value. Overall, our findings support linked TEA as a durable and functionally reliable option for complex distal humeral fractures in carefully selected elderly patients.

## 5 Conclusions

TEA demonstrates durable long-term effectiveness for treating complex distal humeral fractures in elderly patients. At long-term follow-up, TEA demonstrated stable ROM recovery, reliable pain relief, and acceptable complication rates independent of common comorbidities suggesting that TEA represents a valuable primary option in carefully selected geriatric patients. Future multicenter prospective studies incorporating formal survival analysis are warranted to further define implant longevity beyond 10 years and refine patient selection criteria.

## Data Availability

The datasets presented in this article are not readily available because of ethical, legal, or privacy restrictions. Requests to access the datasets should be directed to the corresponding author.
